# Virtual RAMPPS: A Virtual Teaching Method Inspired From the RAMPPS Model

**DOI:** 10.1192/bjo.2024.332

**Published:** 2024-08-01

**Authors:** Lijna Harris Ali, Ramy Teama, Kim Ooi

**Affiliations:** 1 RCPsych, Huddersfield, United Kingdom; 2 RCPsych, North Yorkskire, United Kingdom; 3 RCPsych, York, United Kingdom

## Abstract

**Aims:**

RAMPPS (Recognising and assessing medical problems in Psychiatric settings) training was set up over a decade ago by the then Health Education Yorkshire and Humber Task Group of Clinical skills project workers. Main aim was to address the Physical health agenda in mental health. It was felt that the clinical and support staff in psychiatric settings lacked confidence in recognising and managing physical health issues early on, possibly due to inadequate training in this area. RAMPPS course was designed to address this gap. The course is set up as a face to face multidisciplinary, interprofessional simulation based training with simulated actors, mannequins and other hybrid teaching models .Like any such training, there is a constant need for resources, mannequins, simulated actors, space and funding which could limit the extensive use of this training. We adapted this face to face teaching model for virtual audience to deliver an interprofessional interactive adaptable teaching module using realistic scenarios.

**Methods:**

We adapted some of the scenarios from the RAMPPS module which suited the virtual audience and incorporated into Power point presentation and using an interactive teaching software called Slido we developed Virtual RAAMPPS.

Conducted a few trial sessions within the team and later produced a sample scenario and presented to the medical education team at the trust. The main teaching is the interactive discussion whilst going through the scenarios allowing an impact as close to a face to face teaching as possible.

Next is to do a PILOT Virtual RAMPPS morning session delivered to a group of trainees (psychiatry and foundation trainees) and gather detailed feedback and continue to deliver the pilot teaching a few more times at other avenues and continuously modify the teaching based on the feedback.

**Results:**

Collecting Qualitative feedback from PILOT conducted.

**Conclusion:**

The aim is not to replace simulation based face to face training, but to provide a near enough realistic virtual experience of real life scenarios and to think through them in a systematic and structured way thus enabling better management of some of the physical health dilemmas faced in our psychiatric settings.

It provides the multidisciplinary staff a functional working knowledge of common physical health conditions and its complications encountered in psychiatric setting. The teaching can be modified based on the audience by changing the scenarios relevant to that area of clinical practise or of the patient group.

Allowing anonymity in responses provides a non-judgemental and safe place to make mistakes and eventually improving patient safety and staff experience within Psychiatry.

